# Impact of impaired cerebral blood flow autoregulation on cognitive impairment

**DOI:** 10.3389/fragi.2022.1077302

**Published:** 2022-12-02

**Authors:** Shaoxun Wang, Chengyun Tang, Yedan Liu, Jane J. Border, Richard J. Roman, Fan Fan

**Affiliations:** Department of Pharmacology and Toxicology, University of Mississippi Medical Center, Jackson, MS, United States

**Keywords:** cognitive impairment, cerebrovascular dysfunction, CBF autoregulation, myogenic response, functional hyperemia, capillary abnormalities

## Abstract

Although the causes of cognitive impairment are multifactorial, emerging evidence indicates that cerebrovascular dysfunction plays an essential role in dementia. One of the most critical aspects of cerebrovascular dysfunction is autoregulation of cerebral blood flow (CBF), mainly mediated by the myogenic response, which is often impaired in dementia individuals with comorbidities, such as diabetes and hypertension. However, many unsolved questions remain. How do cerebrovascular networks coordinately modulate CBF autoregulation in health and disease? Does poor CBF autoregulation have an impact on cognitive impairment, and what are the underlying mechanisms? This review summarizes the cerebral vascular structure and myogenic (a three-phase model), metabolic (O_2_, CO_2_, adenosine, and H^+^), and endothelial (shear stress) factors in the regulation of CBF; and the consequences of CBF dysautoregulation. Other factors contributing to cerebrovascular dysfunction, such as impaired functional hyperemia and capillary abnormalities, are included as well. Moreover, this review highlights recent studies from our lab in terms of novel mechanisms involved in CBF autoregulation and addresses a hypothesis that there is a three-line of defense for CBF autoregulation in the cerebral vasculature.

## Introduction

Alzheimer’s disease (AD) and vascular dementia are the most common types of dementia, among which AD accounts for 2/3 of dementia cases (O'Brien and Thomas, 2015). In the United States alone, AD afflicts 6.5 million people, which accounts for 10.7% of individuals over 65 years of age, and this number is estimated to double by 2050 (Gaugler et al., 2022). Dementia disproportionately affects females, who are nearly twice as likely as males to be diagnosed with AD in both middle and old age (Gaugler et al., 2022) (Mazure and Swendsen, 2016). The incidence of AD is greater in age-matched women compared to men (Shekhar et al., 2017b). For example, 81.7% of females versus 24% of males over 85 years old develop AD (Andersen et al., 1999; Gaugler et al., 2022).

The causes of cognitive impairment with aging are multifactorial. Cerebral vascular dysfunction is a major contributor to the development of vascular dementia (Gladman et al., 2019; Iadecola et al., 2019). AD is characterized by extracellular beta-amyloid (Aβ) aggregation in senile plaques and intracellular phosphorylated tau (p-tau) that forms neurofibrillary tangles (Murphy and LeVine, 2010). Therefore, AD is traditionally considered a neurodegenerative disease precipitated by the overproduction of Aβ and p-tau. However, they are not the only factors that contribute to the onset and development of AD, given the fact that current treatments and clinical trials for AD targeting the synthesis and clearance of Aβ and p-tau failed (Zlokovic, 2005; Murphy and LeVine, 2010; Wang et al., 2016; Fang et al., 2022b). Cerebral macro and microvascular dysfunction are commonly presented in AD patients (Iadecola and Gottesman, 2018), which raises the question of whether cerebrovascular dysfunction contributes to the onset and development of AD. In this regard, a recent study demonstrated that cerebral vascular dysfunction precedes by 2-month, Aβ accumulation and cognitive impairment in a TgF344-AD rat model that expresses mutant human amyloid precursor protein and presenilin 1 genes (Fang et al., 2020). AD is closely associated with CBF dysregulation, blood-brain barrier (BBB) breakdown, and neurovascular coupling dysfunction (Kisler et al., 2017; Sweeney et al., 2018). Human studies suggest that cerebrovascular dysfunction, abnormal angiogenesis, and CBF dysregulation might occur even before Aβ deposition, neuronal dysfunction, and grey matter atrophy (Iturria-Medina et al., 2016; Kisler et al., 2017). In addition, cerebral amyloid angiopathy, characterized by the buildup of Aβ in the wall of the cerebral vasculature that causes vascular damage, was reported in ∼80% of postmortem AD cases (McLauchlan et al., 2017; Nyúl-Tóth et al., 2020), suggesting cerebrovascular diseases may contribute to the onset and progression of AD (Iadecola and Gottesman, 2018).

The adult human brain accounts for only 2% of total body mass, while it consumes 20% of cardiac output (Cipolla, 2009). Although the brain is a highly energy-demanding organ, it has limited oxygen and glucose reserves (Cipolla, 2009). Therefore, precise CBF regulation is necessary to ensure normal neuronal function. Impaired CBF autoregulation can cause cerebral ischemic or hemorrhagic injuries that have been associated with dementia in the elderly (Brickman et al., 2015; Shekhar et al., 2017a). Compelling preclinical data have demonstrated that even mild impairment of CBF autoregulation may lead to neuronal death and dementia in the elderly (Tarumi et al., 2014; Kisler et al., 2017; Toth et al., 2017).

CBF autoregulation and functional hyperemia are two critical mechanisms in the modulation of CBF by the coordinate interactions of cerebral arteries, arterioles, and neurovascular coupling in the brain. Neurovascular dysfunction in dementia has been extensively reviewed by peers (Iadecola, 2004; Iadecola and Davisson, 2008; Sweeney et al., 2016; Csiszar et al., 2017; Iadecola, 2017; Kisler et al., 2017; Toth et al., 2017; Iadecola and Gottesman, 2018). This review focuses on the underlying mechanisms of myogenic, metabolic, and endothelial factors that contribute to CBF autoregulation and summarizes recent studies from our lab addressing a new hypothesis of a three-line defense for CBF autoregulation and the potential impact of impaired CBF autoregulation on cognitive impairment. Finally, impaired functional hyperemia and capillary abnormalities are also discussed.

## Cerebral blood flow autoregulation

CBF autoregulation is an essential homeostatic mechanism to maintain a constant CBF to maintain oxygen and glucose delivery to the brain despite fluctuations in cerebral perfusion pressure (CPP) (Fan et al., 2014; Fan et al., 2015; Fan et al., 2016). It is mainly mediated by the myogenic response (MR), an intrinsic property of vascular smooth muscle cells (VSMCs), which allows vasoconstriction in response to elevations in CPP. The response to decreases in CPP is largely associated with the release of vasoactive substances to induce vasodilation, to maintain CBF (Osol et al., 2002; Talman and Nitschke Dragon, 2007).

### Cerebrovascular architecture

Blood flow to the brain is provided by two pairs of large arteries, namely the internal carotid and vertebral arteries, that supply the Circle of Willis that forms an anastomotic ring at the base of the brain (Cipolla, 2009). Three pairs of main intracranial arteries branch off from the circle of Willis, forming anterior, middle, and posterior cerebral arteries, respectively. They give rise to smaller pial arteries and arterioles that distribute on the surface of the brain (Cipolla, 2009). Parenchymal arterioles (PAs) take off from the pial arteries and penetrate the brain cortex at right angles, giving rise to precapillary arterioles and capillaries (Iadecola and Davisson, 2008). Postcapillary venules drain the blood from the capillary bed to the big veins. The aging of the venous system is related to cognitive impairment (Molnár et al., 2021; Nyul-Toth et al., 2022). Each PA supplies a distinct region of the cerebral cortex, which is the “bottleneck” to control downstream CBF (Nishimura et al., 2007). Blockade of even one PA leads to ischemic damage in a capillary bed resulting in functional consequences (Shih et al., 2013).

### Cerebrovascular structure

The wall of the cerebral artery and arterioles are divided into three concentric layers (Cipolla, 2009). The inner layer is the tunica intima, which consists of a concentrated internal elastic lamina (IEL) sheet and a layer of functional endothelial cells. The middle layer is tunica media packed with VSMCs separated by elastin fibers, collagen, and other extracellular matrix (ECM) components. The outer layer is the tunica adventitia, which has longitudinally arranged collagen fibers, fibroblasts, fibronectin, and other ECM proteins. Cerebral arteries lack an external elastic lamina layer, which is different from similar-sized vessels in other vascular beds (Cipolla, 2009). VSMCs are abundant in the wall of cerebral arteries and are closely packed into multiple layers, but PAs only have a single layer of VSMCs. Pericytes are another type of mural cell wrapping around the vascular walls of precapillary arterioles and capillaries. A variety of terminologies have been used to define pericytes on the pre-capillary arterioles by different groups, such as transitional pericytes (Kisler et al., 2017), precapillary smooth muscle cells (Hill et al., 2015), smooth muscle cell-pericytes hybrid (Hartmann et al., 2015), and ensheathing pericytes which are alpha-smooth muscle actin (α-SMA) positive and believed to be the first to respond during functional hyperemia (Grant et al., 2019; Hartmann et al., 2022). The organizational hierarchy and structure of pericytes have been systematically characterized in mice: ensheathing pericytes replace VSMCs at the transition to the arteriole-capillary interface, and mesh pericytes and thin-strand pericytes cover capillaries where there are no VSMCs in the wall (Grant et al., 2019; Liu et al., 2021; Hartmann et al., 2022).

### Sex differences in the structure of the cerebrovasculature

The alteration in cerebrovascular structure has a direct effect on the regulation of CBF. For example, cerebral vascular hypertrophic remodeling in some forms of hypertension reduces cerebrovascular lumen diameter and compliance, which elevates vascular resistance and results in significant CBF hypoperfusion (Faraco and Iadecola, 2013). Previous studies demonstrate that females have a higher basal vascular tone and lower vascular compliance of cerebral arteries compared to males (Muller et al., 1991; Reed et al., 2020). A recent study from our lab demonstrated that middle cerebral arteries (MCAs) isolated from young female Sprague Dawley (SD) rats display smaller inner diameters, thinner wall thickness, reduced vascular distensibility, and increased wall stress compared to age-matched male rats. The MCA of females has fewer layers of VSMCs and an increased level of collagen in the tunica media layer in association with a thicker IEL and reduced numbers and areas of IEL fenestrae than males (Wang et al., 2020b). The range of CBF autoregulation was shifted to lower pressures in females. Nevertheless, there were no sex differences in functional hyperemia and short-term learning and memory in young SD rats (Wang et al., 2020b). However, postmenopausal females are at higher risk for cerebrovascular diseases (such as stroke and vascular dysfunction) related to cognitive impairment in clinical studies (Robison et al., 2019). Although sex differences in MCA structure have not been studied in elderly females, they may contribute to a higher incidence of cerebrovascular diseases and dementia in females with aging.

### Arterial myogenic behavior

As the resistance of arteries and arterioles is proportional to the 4th power of the radius, even small alterations in vascular diameter would be expected to have a dramatic effect on CBF (Lidington et al., 2018). Under normal physiological conditions, CBF is constant due to an autoregulatory mechanism that adjusts vascular diameter in response to fluctuations in blood pressure. Although the regulation of CBF is multifactorial, extrinsic neuronal and hormonal mechanisms, intrinsic metabolic and myogenic mechanisms, and arterial myogenic behavior are all accepted to play a major role in the regulation of CBF (Roman, 2002; Cipolla, 2009).

**FIGURE 1:**
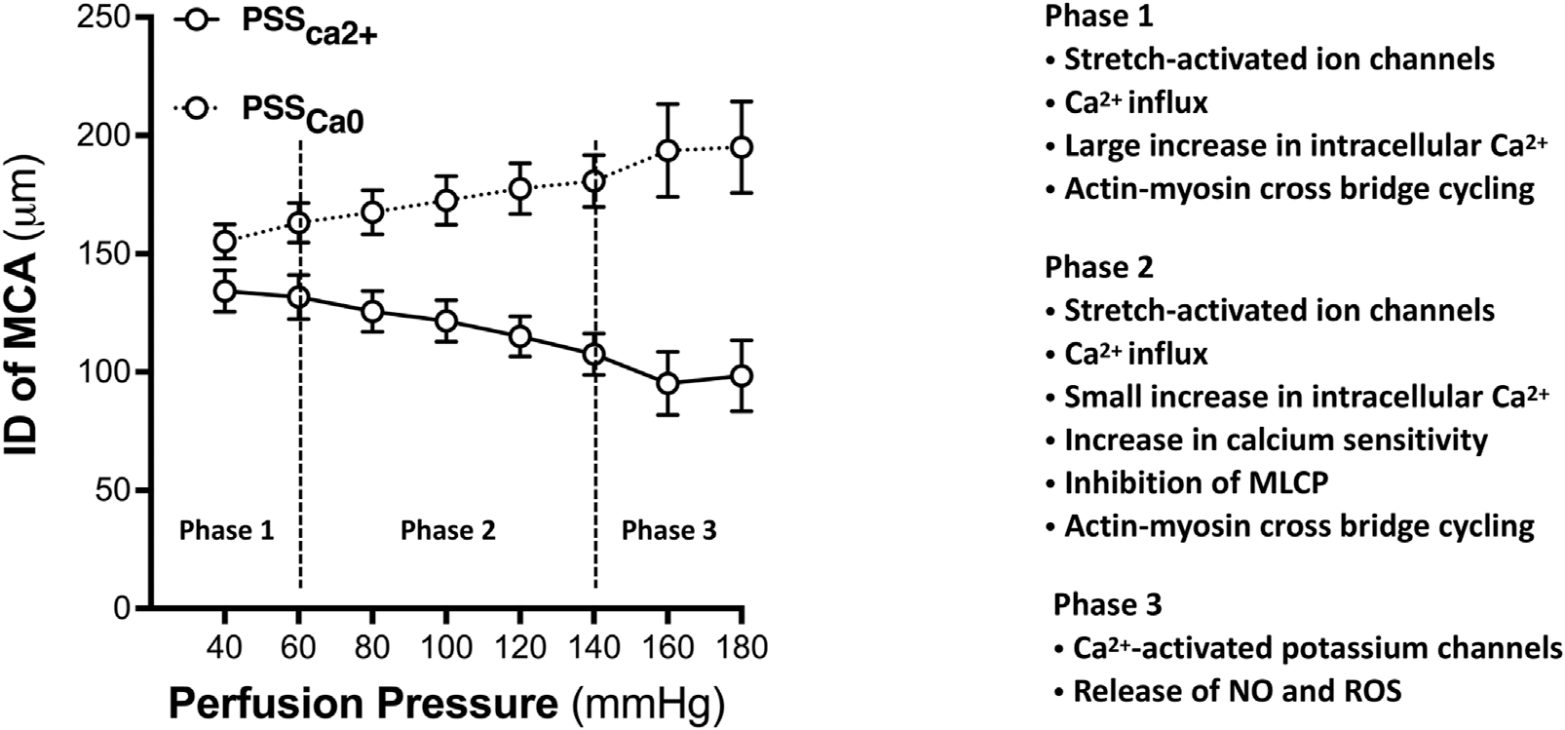
A three-phase model of myogenic behavior of the MCA. Phase 1: the myogenic tone develops at the pressures approximately 40 mmHg in the MCA freshly isolated from 3-month-old healthy male SD rats. During this phase, the MCA constricts relative to its passive distension state. Generation of the myogenic tone is initiated by pressure-induced activation of mechanosensors, which transduces into subsequent biological signaling events resulting in a large increase in intracellular calcium concentrations that promote actin-myosin cross-bridge mediated vasoconstriction. Phase 2: The myogenic response is generated at pressure from 60 to 140 mmHg. In this phase, only a small change in the diameter of the MCA with a slight increase of intracellular Ca2+ despite a significant increase in contractile force. Calcium sensitivity plays a vital role in this phase. Phase 3: Forced dilatation occurs when pressure exceeds 140 mmHg. This phase displays active vasodilation associated with the activation of Ca2+-activated potassium channels. PSSCa2+ represents the active curve of myogenic response when vessels in PSS solution with Ca2+. PSSCa0 represents the passive curve when vessels in PSS solution without Ca2+. Data are presented as mean values ± SEM. The MCAs were obtained from 5 SD rats. PSS, Physiological salt solution; ID, Inner diameter; MCA, Middle cerebral artery; GPCRs, G-protein-coupled receptors; MLC20, 20 kDa Myosin light chain; CaM, Calmodulin.

A century ago, Sir William Bayliss observed vasoconstriction following an elevation in transmural pressure in arteries in dogs, cats, and rabbits (Bayliss, 1902). This physiological phenomenon was later shown to be a generalized intrinsic property of VSMCs in resistance arteries and arterioles. The arterial myogenic behavior can be generally divided into three phases based on the pressure range (Osol et al., 2002). In the first phase (Figure 1), the vessel initially develops the myogenic tone relative to its passive distension state. In the cerebral circulation, this phase commonly can be found at intraluminal pressures of 40–60 mmHg. As intraluminal pressure is increased further to 60–140 mmHg, the second phase of the myogenic response (MR) occurs. In this phase, the vessels constrict as pressure is elevated. Autoregulation range is the blood pressures between which the brain can maintain a constant CBF. When transmural pressure exceeds the autoregulatory range, typically above 140 mmHg, the vessels exhibit distension. This stage is the third phase and termed forced dilatation.

The development of myogenic tone is initiated by pressure-induced activation of mechanosensors due to mechanical stimuli-caused deformation of VSMCs and increased wall tension. Multiple potential mechanosensors have been identified, including, but not limited to, stretch-activated ion channels, G-protein coupled receptors (GPCRs), integrins, cadherins, and membrane-bound tumor necrosis factor (Kroetsch et al., 2017; Lidington et al., 2018; Hong et al., 2020). The initial mechanical stimuli phase is characterized by a significant increase in intracellular calcium and triggers subsequent biological signaling events. Underlying mechanisms involving calcium influx in the 1^st^ phase of the arterial myogenic behavior include the opening of stretch-activated ion channels, such as transient receptor potential (TRP) channels and epithelial sodium (ENaC) channels (Kim et al., 2013); reorganization of the cytoskeleton (Davis et al., 2001); activation of the diacylglycerol (DAG)—phospholipase C (PLC)-protein kinase C (PKC) signaling pathway (Hong et al., 2020); activation of the PLC—inositol 1,4,5-trisphosphate (IP3) signaling pathway and the stimulation of calcium release from the sarcoplasmic reticulum (Hong et al., 2020). As a consequence of increased calcium entry in VSMCs, calcium-calmodulin activates myosin light chain kinase (MLCK), subsequently phosphorylating the 20 kDa myosin light chain (MLC_20_), resulting in increased cross-bridge formation and VSMC constriction. The activation of mechanosensors in response to an elevation in pressure induces VSMCs depolarization and subsequently triggers the opening of voltage-gated activated calcium channels, L-type calcium channels, and inhibition of calcium-activated potassium channels (K_ca_).

The MR refers to vasoconstriction in response to an increase in transmural pressure or vasodilation to a decrease in pressure. In the cerebral circulation, when intraluminal pressure is in the range of 60–140 mmHg, the myogenic tone has already been generated, and a cascade of signaling events promoting calcium influx has already been established. Nevertheless, only a small increase in intracellular calcium (Ca^2+^) was found, and there was a limited diameter change in this phase (Osol et al., 2002). Although elevation in intracellular Ca^2+^ concentration is necessary for the initiation of myogenic constriction, it is the sensitivity of the contractile element to Ca^2+^ that determines the constriction force of VSMCs at a constant Ca^2+^ intracellular level (Moreno-Domínguez et al., 2013). Thus, calcium sensitization, a calcium-independent mechanism, also plays a major role in the regulation of MR in addition to calcium-dependent mechanisms (Osol et al., 2002; Touyz et al., 2018). Many mechanisms have been reported to regulate calcium sensitization. However, the regulation of the phosphorylation of MLC_20_ is calcium-calmodulin-MLCK independent. Major signaling pathways and molecular targets that have been reported in calcium sensitization include the regulation of myosin phosphatase (Somlyo and Somlyo, 2003; Abd-Elrahman et al., 2015); the activation of the diacylglycerol-phospholipase C-protein kinase C (DAG-PLC-PKC) signaling pathway (Osol et al., 1991; Schubert et al., 2008); the RhoA-Rho kinase pathway (Schubert et al., 2008; Loirand and Pacaud, 2010); the rearrangement of the actin cytoskeleton and thin filament (Cipolla and Osol, 1998; Veerareddy et al., 2004); and increased production of reactive oxygen species (ROS) (Schubert et al., 2008). Additionally, integrin-linked kinase, zipper-interacting protein kinase, and p21-activated protein kinase have also been implicated in the regulation of calcium sensitization (Deng et al., 2001; Endo et al., 2004). Extracellular-signal-regulated kinase (ERK) regulates the function of caldesmon and inhibits the interaction of actin-myosin resulting in decreased calcium sensitivity of the contractile mechanism (Kordowska et al., 2006).

When pressure exceeds the upper limit of the CBF autoregulatory range of nearly 140 mmHg, forced dilatation occurs. In this phase, myogenic tone is lost, and there is a rapid increase in stress on the cell membrane and vessel wall tension, resulting in significant increases in intracellular calcium concentration in endothelial cells. This phase is associated with the activation of the K_Ca_ channel (Paterno et al., 2000) and dynamic changes of the actin cytoskeleton with depletion of G-actin (Cipolla and Osol, 1998). Additionally, the integrity of the vascular structure seems to be important in this phase. Our recent study demonstrated that the forced dilatation occurred at lower pressures in young, healthy females than in age-matched male rats (Wang et al., 2020b), indicating sex differences in the structure and function of rat MCA may play a role in the onset and development of cerebral vascular disease and dementia, especially upon aging. Moreover, vascular structural changes and damage, such as hypertrophy, hyaline degeneration, and fibrosis of the vessel wall seen in some models of hypertension, also shifted the forced dilatation to lower pressures resulting in poor CBF autoregulation and downstream pathological consequences (Strandgaard et al., 1975). In contrast, other studies demonstrated that CBF autoregulation is shifted to higher pressures in spontaneously hypertensive rats and other models of chronic hypertension (Sadoshima et al., 1985; Cipolla, 2007). Such differences in the autoregulatory response in hypertension may be due to different genetic backgrounds, preparations, and regulatory mechanisms.

The Shih group’s recent study that identified mouse pericytes localized on the wall of the 1st to 4th order of pre-capillary arterioles, termed ensheathing pericytes, are α-SMA positive (Grant et al., 2019; Hartmann et al., 2022) accelerated the search to the answer whether a subgroup of pericytes that expresses α-SMA also regulates CBF. It has been well established that VSMCs play a vital role in the regulation of arterial myogenic reactivity and CBF autoregulation, and our lab revealed that high glucose-treated pericytes showed diminished contractile capability. Furthermore, an impaired myogenic response of PA and poor CBF autoregulation in the deep cortex of DM rats was also observed (Liu et al., 2020). However, whether contractile properties of cerebral vascular pericytes contribute to the regulation of CBF remains in controversy and it can only be explained by the existence of several subtypes of pericytes, which are heterogeneous in their morphology and function (Armulik et al., 2010).

### Vascular response to shear stress

While mechanical forces induce morphological and functional changes in VSMCs (and possibly pericytes) to alter arterial myogenic behavior and trigger a set of subsequent biochemical and biological events, frictional force causes endothelial shear stress. Shear stress is defined as a tangential force on the endothelium generated by flow moving within the vasculature proportional to blood flow (Sriram et al., 2016). Sustained blood flow with high laminar shear stress promotes endothelial survival and quiescence, but reduced shear stress with turbulent flow promotes endothelial apoptosis, vasoconstriction, and coagulation (Paszkowiak and Dardik, 2003).

Shear stress induces vasodilation in systemic vessels through the release of endothelial-dependent vasodilators, such as nitric oxide (NO) (Thorin-Trescases and Bevan John, 1998), prostaglandins (Koller et al., 1993), prostacyclin (PGI_2_) (Okahara et al., 1998), and epoxyeicosatrienoic acids (EETs) (Sun et al., 2007), *via* complex signal transduction pathways that involve mechanosensor activation and enzymatic reactions (Sriram et al., 2016). Multiple mechanosensors have been proposed to initiate shear stress-related signaling transduction in endothelial cells, including ion channels, GPRs, a hyaluronan receptor CD44, extracellular adenosine, integrins, and cytoskeleton remodeling (Li et al., 2010; Sriram et al., 2016; DeOre et al., 2020). The vascular signals are then retrogradely propagated along the endothelium for vasomotor responses. NO released from endothelial cells diffuses into VSMCs, leading to activation of soluble guanylyl cyclase (sGC) and cyclic guanine monophosphate (cGMP). As a second messenger, cGMP activates protein kinase G, which opens potassium channels to induce VSMCs hyperpolarization and vasodilation. There is also evidence that the response to NO in small cerebral arterioles is cGMP independent and is associated with inhibition of the formation of 20-hydroxyeicosatetraenoic acid (20-HETE) (Sun et al., 1998; Hall et al., 2014).

### Cerebral blood flow autoregulation: A three-line of defense?

**FIGURE 2:**
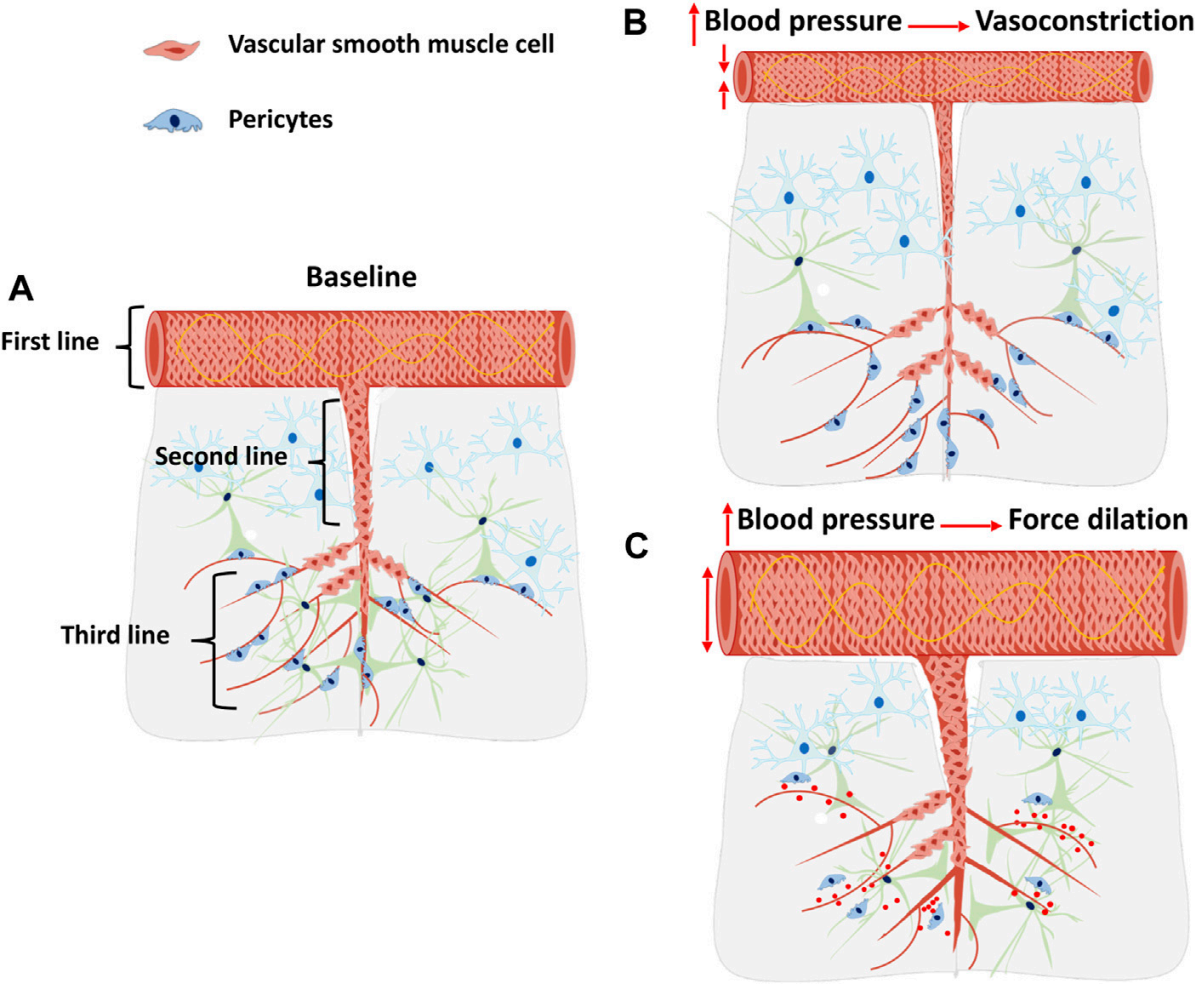
CBF Autoregulation: A three lines of defense?, A cartoon illustrates the three lines of defense of CBF autoregulation and the consequences of impaired CBF autoregulation. **(A)** Cerebral pial arteries and arterioles at a basal level under baseline blood pressure. **(B)** Cerebral pial arteries and arterioles constrict in response to elevated blood pressure. **(C)** Cerebral pial arteries and arterioles force dilated in response to elevated blood pressure due to an impaired myogenic response. The impaired CBF autoregulation may contribute to pericytes detachment, capillaries damage, microvessels rarefaction, and blood-brain barrier breakdown.

CBF autoregulation maintains a constant CBF to consistently maintain oxygen and glucose delivery to the brain despite the fluctuation of cerebral perfusion pressure (CPP) (Fan et al., 2015). It is mainly mediated by MR, an intrinsic property of VSMCs, which allows vasoconstriction in response to elevations in CPP. The response to decreases in CPP is largely associated with the release of vasoactive substances to induce vasodilation and maintain CBF (Osol et al., 2002; Talman and Nitschke Dragon, 2007). Increases in segmental vascular resistance limit perfusion pressure from being transmitted to downstream capillaries, preventing rupture and cerebral vasogenic edema. Unlike most peripheral vascular beds in which precapillary arterioles contribute the most to vascular resistance, in the cerebral circulation, MCAs and pial arteries account for approximately 50% of vascular resistance. Downstream PAs and capillaries contribute to the remaining cerebral vascular resistance (Shapiro et al., 1971; Faraci and Heistad, 1990). CBF autoregulation is primarily regulated by MR occurring at different levels of the vascular networks, including pial arteries, PAs, and precapillary arterioles (Figure 2). These cerebral arteries and arterioles coordinately regulate CBF autoregulation to protect downstream capillary integrity and BBB that prevents the brain from ischemic or hemorrhagic injury. It is widely accepted that the primary contributors to the MR are VSMCs that enwrap the wall of arteries and arterioles. However, emerging recent evidence suggests that α-SMA positive pericytes, a subtype of mural cells that are embedded in the cerebral capillary basement membrane and wrap around the endothelial cells on precapillary arterioles and capillaries, also actively regulate cerebral vascular resistance, which may provide a novel insight into mechanisms of CBF autoregulation at arterioles and capillaries levels (Hall et al., 2014; Hill et al., 2015; Watson et al., 2020).

CBF autoregulation on the surface of the brain is regulated by the MR of the pial arteries and the MCA, which could be considered the first line of defense of cerebral circulation (Fan et al., 2015). MCA isolated from young, healthy male SD rats with intact MR constricts in response to elevated transmural pressure at the range of 40–160 mmHg (Pabbidi et al., 2018). However, surface CBF autoregulation is inadequate to prevent increased CPP from being transmitted to downstream arterioles and capillaries when the blood pressure exceeds the surface CBF autoregulatory range above 140–160 mmHg or under pathological conditions. Hence, a second line of defense is needed to protect downstream microvessels.

The PA serves as the “bottleneck” of cerebral circulation, limiting elevated CBF and CPP from reaching downstream capillaries (Nishimura et al., 2007; Cipolla et al., 2014). PAs have a higher myogenic tone at low pressures than the MCAs and pial arteries due to increased L-type voltage-dependent calcium channels activity and uncoupled large-conductance calcium-activated potassium channels (BK_Ca_) (Cipolla et al., 2014), which largely affects cerebral perfusion (Pires et al., 2015; Zhang et al., 2020). Isolated PAs from young SD rats constrict by 20% in response to transmural pressure increases from 10 to 30 mmHg (Zhang et al., 2020). Therefore, MR of the PAs, which actively regulates CBF in the deep cortex, serves as the second line of defense for CBF autoregulation. A recent study demonstrated that deep cortical CBF at the capillaries level increased to a greater extent than surface CBF in response to an acute elevation in blood pressure in normal SD rats, which suggests that constriction of PAs blunts transmission of pressure from the pial circulation to the deep cortical capillaries (Wang et al., 2020a). The loss of MR of the MCA and PA seen in aged diabetic (DM) animals lead to impaired surface and deep cortical CBF autoregulation, which contribute to BBB leakage, tight junction breakage, pericyte detachment, and microvessel rarefaction in the CA3 region in the hippocampus. Such cerebrovascular dysfunction could result in inflammation, neurodegeneration, and cognitive impairment in aged DM rats (Wang et al., 2020a).

The functions of cerebrovascular mural cells in regulating CBF at different vascular beds are remained controversial due to their heterogeneities. Contractility of precapillary VSMCs, rather than capillary pericytes, was thought to play a dominant role in the regulation of deep cortical CBF. This opinion was based on the observation that pericytes completely lack the expression of contractile protein α-SMA and that precapillary arterioles are covered by α-SMA positive VSMCs with a circumferential band-like morphology (Hill et al., 2015). Single-cell RNA sequencing showed pericytes have low levels of contractile markers including ACTA2 and MYL9. However, there is a subcluster of cerebrovascular mural cells that co-express ACTA2, PDGFRB, and RGS5, suggesting these cells may be contractile pericytes (Gastfriend et al., 2021). Contrarily, other reports indicated that microvascular pericytes in the adult mouse cortex exhibit structural diversity based on their localization. Ensheathing pericytes that enwrap around the precapillary arterioles with ovoid cell bodies are positively expressed α-SMA (Hartmann et al., 2015; Grant et al., 2019). Although no direct evidence has yet to indicate whether ensheathing pericytes are contractile or whether these cells can cause precapillary arterioles constriction in response to elevated CPP to regulate CBF, a recent study demonstrated that a mild deficiency of pericytes caused an increase in red blood cell velocity in precapillary arterioles and capillaries (Watson et al., 2020), suggesting that pericytes contribute to cerebral vascular resistance. Moreover, Hall et al. (2014) found that isolated capillary pericytes are contractile, and these pericytes can induce capillary constriction or dilation in response to stimulation of neurotransmitters like noradrenaline and glutamate. Glutamate activates the NMDA (N-methyl-d-aspartate) receptor to release vasodilator nitric oxide that promotes capillary dilation by inhibiting of 20-HETE production which incites pericytes dilation (Hall et al., 2014). Ischemic injury induces pericytes constriction possibly due to ATP depletion, which cripples ion pumping resulting in increased intracellular Ca^2+^ levels (Hall et al., 2014). Nevertheless, capillary pericytes identified in this study were only based on the vessel size (<10 µm) and neuron-glia antigen 2/platelet-derived growth factor receptor beta (NG2/PDGFRβ) expression without validation by morphological criteria and documentation of α-SMA expression. Vessel size <10 µm also includes precapillary arterioles that contain VSMC-pericyte transition regions covered by contractile VSMCs and ensheathing pericytes, which have heterogeneous α-SMA expression and have contractile capability. Future studies need to be carried out to determine whether pericytes on the precapillary arterioles also have myogenic properties that modulate capillary diameter and CBF autoregulation in response to changes of perfusion pressure, that may form the third line of defense. Further studies need to be carried out to investigate the diverse functions of cerebrovascular mural cells. Single-cell RNA sequencing may be an excellent approach to dissecting the heterogeneities of cerebrovascular mural cells.

### Metabolic control of cerebral vascular tone

Cerebrovascular tone and CBF is also modulated by the local release of metabolic factors from neurons and glial cells to fulfill the energy demand of brain activity (Pi et al., 2018). Many vasoactive metabolites, such as O_2_, CO_2_, adenosine, and H^+^, have been reported to participate in the regulation of CBF (Smith and Ainslie, 2017): an increase in the partial pressure of carbon dioxide (pCO_2_), H^+^, adenosine levels or a decrease in the partial pressure of oxygen (pO_2_) may induce vasodilation and CBF elevation (Smith and Ainslie, 2017).

Since the brain is a highly oxygen-demand organ and oxygen is necessary for aerobic metabolism, it is no surprise that hypoxia modulates vasomotor responses and CBF (Lynch et al., 2006). Cerebral vessels dilate to maintain CBF when brain tissue pO_2_ falls below ∼50 mmHg. It seems that there is an oxygen sensor in the vessel wall that acts independently of pCO_2_ and H^+^ (Busse et al., 1984; Frisbee et al., 2002; Lynch et al., 2006). When a decreased pO_2_ is sensed by the skeletal muscle, dilation occurs mediated by endothelial-dependent NO release in mild hypoxia, in combination with decreased 20-HETE and increased PGI_2_ in moderate hypoxia, and by the release of PGI_2_ and adenosine in severe hypoxia (Winn et al., 1979; Bari et al., 1998; Frisbee et al., 2002). Although it remains to be determined whether similar mechanisms regulate the hypoxia response in the cerebral circulation, vasodilation in isolated MCAs in response to reduced pO_2_ is associated with the release of endothelium-dependent cyclooxygenase products that activate potassium (K^+^) channels (Fredricks et al., 1994). Another study demonstrated that PGI_2_ is also a major contributor to hypoxia-induced dilation among these hypoxia-related vasoactive mediators (Lombard et al., 1999).

Adenosine is a purine nucleoside produced from ATP metabolism. It is a potent vasodilator in cerebral circulation and an active mediator to increase CBF in response to hypoxia, ischemia, reduced blood pressure, and functional hyperemia (Lynch et al., 2006). Adenosine is an endothelium-derived hyperpolarizing factor (EDHF) (Ohta et al., 2013). It binds adenosine 2a receptor (A_2a_R) or 2b receptor (A_2b_R) to activate adenylate cyclase and form cAMP that opens K^+^ channels leading to VSMCs hyperpolarization and vasodilation (Edvinsson and Fredholm, 1983; Hwa et al., 2000). A preclinical study demonstrated that knockout or blocking of A_2a_R and A_2b_R significantly impaired CBF autoregulation under hypotension in mice (Kusano et al., 2010). Other studies indicated that adenosine is required not only in response to cerebral hypoxia but also in maintaining basal CBF under normoxic conditions (Frisbee et al., 2002). The blockade of adenosine receptors results in an approximately 20% reduction in CBF under normoxic conditions (Willie et al., 2014).

CO_2_ is an essential regulator of CBF. Elevations in pCO_2_ resulting from increased metabolic activity and the accumulation of CO_2_ causes profound vasodilation and an increase in CBF (Yoon et al., 2012). A 1 mmHg increase in pCO_2_ causes approximately a 3%–6% increase in CBF. Inhalation of 5%–10% CO_2_ increases CBF by 50%–250% in animals and humans (Kety and Schmidt, 1948; Jackson et al., 1974; Tuteur et al., 1976). It is widely accepted that alterations in pCO_2_ accompany changes in pH to regulate cerebrovascular tone and CBF. An increase in pCO_2_ in conjunction with a decrease in pH leads to vasodilation. Similarly, a decrease in pCO_2_ with an increase in pH leads to vasoconstriction (Yoon et al., 2012). This is evidenced by acidic hypercapnia, but not by isohydric hypercapnia, which induced vasodilation in arterioles and precapillary microvessels (Yoon et al., 2012). Additionally, changes in pH can regulate cerebral arteriolar tone at a constant pCO_2_ level, indicating that pH has independent effects on vascular tone (Kontos et al., 1977; Dabertrand et al., 2012). The view that pCO_2_ itself regulates vascular tone independent of pH is supported by the study demonstrating that a potent constriction was induced upon lowering pCO_2_ at a constant pH of 7.4 in isolated cat MCA, and vasodilation occurred in response to elevation of pCO_2_ at a constant pH level (Harder and Madden, 1985). CO_2_/pH can also act directly on vascular endothelial cells to promote the release of vasoactive factors, including NO, endothelin-1, and EDHF (Yoon et al., 2012) to alter intracellular Ca^2+^ levels in VSMCs (Edvinsson and Sercombe, 1976; Harder and Madden, 1985).

### Novel mechanisms involved in cerebral blood flow autoregulation

Impaired CBF autoregulation has been found in aging and multiple pathological conditions such as chronic DM, hypertension, and obesity (Abd-Elrahman et al., 2015; Toth et al., 2015; Sauve et al., 2016; Shekhar et al., 2017c; Toth et al., 2017). A variety of novel mechanisms that contribute to the autoregulation of CBF and MR have been addressed during the past years. 20-HETE is a potent vasoconstrictor affecting cerebrovascular MR and CBF autoregulation by inhibiting BK_ca_ channels and activating L-type Ca^2+^ channels, which causes depolarization of VSMCs to induce vasoconstriction (Harder et al., 2011; Roman and Fan, 2018). The reduction in 20-HETE results in impaired cerebrovascular MR and CBF autoregulation, contributing to BBB leakage elevations in blood pressure in Dahl Salt-Sensitive (Dahl S) rats (Fan et al., 2015; Shekhar et al., 2017a; Gonzalez-Fernandez et al., 2021). Similarly, a decrease in 20-HETE production with aging contributes to the loss of CBF autoregulation in angiotensin II (Ang II)-induced hypertensive mice (Toth et al., 2015).

γ-Adducin, a cytoskeletal protein, is an actin-capping protein regulating the dynamics of the actin cytoskeleton (Fan et al., 2017a; Fan et al., 2020). Loss of γ–adducin results in excess actin polymerization, leading to the overproduction of abnormal branched F-actin that replaces the stress fibers within the cell body of VSMCs and disrupts signal transduction and membrane trafficking (Fan et al., 2020). Downregulation of γ-adducin impairs both CBF and renal blood flow autoregulation due to impaired MR associated with enhanced BK_ca_ channel activity in VSMCs (Fan et al., 2017a; Fan et al., 2020).

DM causes an imbalance of mitochondrial dynamics in cerebral VSMCs and α-SMA positive pericytes, characterized by increased mitochondrial fission and decreased fusion, resulting in elevated ROS production, adenosine triphosphate (ATP) depletion, and diminished contractile capability of cerebral VSMCs and pericytes (Guo et al., 2020; Liu et al., 2020). These findings may provide novel insights into the understanding of mechanisms by which long-standing DM causes impaired MR and CBF autoregulation and may contribute to cognitive impairment.

Matrix metallopeptidase 9 (MMP9), a member of matrix metalloproteinases, regulates extracellular matrix deposition. An elevation of MMP9 has been reported to accompany BBB leakage and vasogenic edema following ischemic stroke, which leads to cognitive impairment (Weekman and Wilcock, 2016; Underly and Shih, 2021). However, little is known about whether MMP9 is involved in the regulation of cerebrovascular MR and CBF autoregulation. Fan et al. (2017b) reported that knockout of MMP9 in Dahl S rats rescues the development of cognitive impairment after the induction of hypertension, possibly by attenuating cerebrovascular remodeling, improving cerebrovascular MR and CBF autoregulation. To date, the underlying mechanisms have not been determined, and further studies are needed.

### Consequences of impaired cerebral blood flow autoregulation

Poor CBF autoregulation has been reported in patients with mild cognitive impairment or frank dementia (Marshall, 2012). Given the fact that DM, hypertension, and obesity are the primary risk factors for dementia, especially with aging, and they all exhibit dysfunction of cerebral hemodynamics (Shekhar et al., 2017a; Wang et al., 2017; Shekhar et al., 2019), pathological consequences of impaired CBF autoregulation may contribute to the onset and development of cognitive impairments. Loss of CBF autoregulation may restrict the blood flow delivered to ischemic regions after ischemic stroke or traumatic brain injury, leading to a more severe ischemic injury to the brain tissue and neurons (Jordan and Powers, 2012; Armstead, 2016; Shekhar et al., 2017a; Shekhar et al., 2019). It also could transmit excess pressure to downstream capillaries, leading to disruption of capillary integrity and BBB breakdown (Shekhar et al., 2017a; Wang et al., 2017). Disruption of BBB has been reported to cause vasogenic edema, microvessel refraction, and microhemorrhage, all of which contribute to inflammation, glial cell activation, neuronal damage, or neurodegeneration (Bell and Zlokovic, 2009), and are hallmarks of cognitive deficits in both AD (Donev et al., 2009) and vascular dementia (Iadecola, 2013).

## Other mechanisms related to cerebrovascular dysfunction

### Functional hyperemia

Functional hyperemia is the response of the brain in which local increases in neuronal activity trigger elevations in blood flow to meet the brain’s metabolic needs independent of changes in blood pressure. It is mediated by the interactions of a group of cells called the neurovascular unit and includes neurons, astrocytes, endothelial cells of BBB, VSMCs, pericytes, and extracellular matrix (Toth et al., 2017). Capillary flow is characterized by heterogeneity at rest which holds space for blood redistribution during functional hyperemia (Hartmann et al., 2022). Pericytes have been shown to be able to constrict or dilate microvasculature, suggesting the possibility of pericytes in regulating vascular responses to neurotransmitters, changes in neuronal activity, or electrical stimulation (Attwell et al., 2010; Kisler et al., 2017; Kaplan et al., 2020). Furthermore, ensheathing pericytes at arteriole-capillary transition are found to play a major role in fine-tuning the control of functional hyperemia along penetrating arterioles by preferentially dilating specific branches according to the direction of the incoming conductive signals (Gastfriend et al., 2021; Hartmann et al., 2022). Previous studies indicated that local mediators (NO, PGs, and EETs) are released from neurons and/or astrocytes trigger neurovascular coupling to dilate VSMCs in PAs and to pial arteries upon neuronal activation (Longden et al., 2017; Wang et al., 2020a; Tarantini et al., 2021b). Blockade of NO, PGs, and EETs has been found to blunt functional hyperemic responses by about 50%. More recently, several investigators have reported that during neuronal activity, K^+^ accumulates externally and activates K^+^ channels of the inwardly rectifying potassium (Kir) 2.1 family in capillary endothelial cells, causing membrane hyperpolarization which is then propagated along endothelial cells *via* gap junctions to upstream arterioles and surface arteries. This hyperpolarization subsequently spreads to neighboring VSMCs *via* gap junctions, deactivating voltage-dependent Ca^2+^ channels, reducing intracellular Ca^2+^ levels, diminishing Ca^2+^-dependent actin-myosin cross-bridge cycling, and inducing vasodilation (Tallini et al., 2007; Segal, 2015; Longden et al., 2017). It has previously been reported that activation of the Kir2.1 channel is an early endothelial response to shear stress (Hoger et al., 2002). Studies using various channel blockers suggest that the Kir2.1 pathway likely mediates at least half of the functional hyperemic response. A recent study from our lab found the functional hyperemic response to whisker stimulation was markedly impaired in both AD and DM animal models (Wang et al., 2020a). Moreover, it has been found that phosphatidylinositol 4,5-bisphosphate (PIP2) is a key regulator of capillary endothelial cell’s Kir2.1 channels, because reduced PIP2 diminishes capillary endothelial cell Kir2.1 activity, which may contribute to impairment of functional hyperemia (Mughal et al., 2021). Notably, a reduction in PIP2 levels has been observed in AD mouse models as amyloid beta peptides induce phospholipase-mediated PIP2 hydrolysis (Strosznajder et al., 1999; Fang et al., 2022a), further disrupting normal functional hyperemia activity and worsening cerebral hypoperfusion. The hope is that PIP2 analogs may emerge as a promising therapeutic approach for treating dementia, although further study is needed to confirm its long-term effectiveness (Mughal et al., 2021). Age-related impairment of functional hyperemia has been reported to be linked with age-related deficiency of insulin-like growth factor-1, which is believed to be vasoprotective and anti-geronic (Tarantini et al., 2021b; Tarantini et al., 2021c). Another study also found that senescent cells are abundant in the brain of aged mouse which might contribute to neurovascular coupling dysfunction and could be a new therapeutic target (Tarantini et al., 2021a).

### Capillary abnormalities

In addition to Aβ accumulation and tau hyperphosphorylation accompanying AD, there has been increasing interest in alterations in the cerebral microcirculation. Intact structural and functional integrity of cerebral capillaries is critical for ensuring normal function of the central nervous system (Tuma, 2008). Capillary abnormalities, including pericyte degeneration, pericapillary fibrosis, and loss of tight junctions, have been observed in aging, hypertension, DM, and AD. All these pathological alterations lead to a defective BBB (Østergaard et al., 2015; Wang et al., 2020a). Temporary interruption of capillary blood flow is termed capillary stalling, which is driven by constriction of actin-containing capillary pericytes or a stalled leukocyte and is believed to contribute to cerebral hypoperfusion leading to cognitive impairment. There are few factors that induce capillary stalling: Increased circulating leukocytes, expression of cell adhesion molecules, and ROS in chronic inflammation (Crumpler et al., 2021), and loss of cerebral capillary endothelial glycocalyx that is involved in the interactions between blood and endothelial cells. Not surprisingly, dementia-associated risk factors often coexist with chronic low-level inflammation. This theory was recently confirmed by a study conducted by Yoon et al. (2022) using two-photon microscopy and *in vivo* optical coherence tomography angiography.

## Conclusion

The mechanisms and causes underlying dementia are extremely complex. Although AD and Alzheimer’s disease-related dementias (ADRD) are commonly believed to be neurodegenerative diseases, emerging evidence has suggested a role for cerebrovascular dysfunction in the pathogenesis of AD and ADRD. The intact cerebrovascular structure is essential for CBF autoregulation, and alterations directly impact cerebrovascular function. Hypertension, a well-known risk factor of dementia, is linked with cerebral vascular hypertrophic remodeling leading to a reduced cerebrovascular lumen diameter and compliance and, ultimately, CBF hypoperfusion. In addition, the increased incidence of females with dementia also implies the sex differences in the structure and function of cerebrovasculature may play a role in the onset and development of dementia, especially upon aging. Arterial myogenic behavior is the driving force of CBF autoregulation. MR can be generally divided into three phases based on pressure range: the first phase is the development of basal myogenic tone at pressures of 40–60 mmHg; the second phase is myogenic reactivity from 60 to 140 mmHg and forced dilation is the third phase when transmural pressure exceeds 140 mmHg. The MR of the pial arteries, PAs, and pericytes on the precapillary arterioles form the three-line of defense for CBF autoregulation. In addition to vascular aspects, metabolic factors also modulate CBF by the local release of mediators from neurons and glial cells to satisfy the brain’s energy needs. Many novel mechanisms involved in impairments of CBF autoregulation, MR, functional hyperemia, and BBB integrity have been proposed, such as amyloid-induced PIP2 hydrolysis, 20-HETE reduction, γ-Adducin downregulation, an imbalance of mitochondrial dynamics in cerebral VSMCs and α-SMA positive pericytes and elevations in MMP9. Loss of CBF autoregulation may limit blood flow to ischemic regions or transmit excessive blood flow and pressure, damaging capillary and BBB integrity following elevations in pressure. Functional hyperemia regulates CBF through a retrograde manner independent of changes in blood pressure to fulfill brain’s metabolic demand during elevations in neuronal activity. Dysfunction of functional hyperemia also contributes to cognitive impairment. Capillary abnormalities are another factor that can contribute to cerebrovascular dysfunction and dementia.
